# PCR Inhibition of a Quantitative PCR for Detection of *Mycobacterium avium* Subspecies *Paratuberculosis* DNA in Feces: Diagnostic Implications and Potential Solutions

**DOI:** 10.3389/fmicb.2017.00115

**Published:** 2017-02-02

**Authors:** Kamal R. Acharya, Navneet K. Dhand, Richard J. Whittington, Karren M. Plain

**Affiliations:** ^1^Sydney School of Veterinary Science, Faculty of Science, University of SydneyCamden, NSW, Australia; ^2^Department of Livestock Services, Regional Veterinary Diagnostic LaboratoryDhangadhi, Nepal; ^3^Sydney School of Veterinary Science, School of Life and Environmental Sciences, University of SydneyCamden, NSW, Australia

**Keywords:** PCR inhibition, qPCR diagnosis, *Mycobacterium paratuberculosis*, relief PCR inhibition, fecal PCR, Johne’s disease (JD) diagnosis

## Abstract

Molecular tests such as polymerase chain reaction (PCR) are increasingly being applied for the diagnosis of Johne’s disease, a chronic intestinal infection of ruminants caused by *Mycobacterium avium* subspecies *paratuberculosis* (MAP). Feces, as the primary test sample, presents challenges in terms of effective DNA isolation, with potential for PCR inhibition and ultimately for reduced analytical and diagnostic sensitivity. However, limited evidence is available regarding the magnitude and diagnostic implications of PCR inhibition for the detection of MAP in feces. This study aimed to investigate the presence and diagnostic implications of PCR inhibition in a quantitative PCR assay for MAP (High-throughput Johne’s test) to investigate the characteristics of samples prone to inhibition and to identify measures that can be taken to overcome this. In a study of fecal samples derived from a high prevalence, endemically infected cattle herd, 19.94% of fecal DNA extracts showed some evidence of inhibition. Relief of inhibition by a five-fold dilution of the DNA extract led to an average increase in quantification of DNA by 3.3-fold that consequently increased test sensitivity of the qPCR from 55 to 80% compared to fecal culture. DNA extracts with higher DNA and protein content had 19.33 and 10.94 times higher odds of showing inhibition, respectively. The results suggest that the current test protocol is sensitive for herd level diagnosis of Johne’s disease but that test sensitivity and individual level diagnosis could be enhanced by relief of PCR inhibition, achieved by five-fold dilution of the DNA extract. Furthermore, qualitative and quantitative parameters derived from absorbance measures of DNA extracts could be useful for prediction of inhibitory fecal samples.

## Introduction

Johne’s disease (JD) is a chronic granulomatous enteric disease of ruminants caused by *Mycobacterium avium* subspecies *paratuberculosis* (MAP) (Sweeney et al., 1992; Dennis et al., 2008). JD control programs have been initiated worldwide, including in the United States, Australia, Japan, and Europe (Kobayashi et al., 2007; Bakker, 2010; Kennedy and Citer, 2010; Whitlock, 2010) as a result of its economic and possible zoonotic significance (Ott et al., 1999; Chiodini et al., 2012). The control strategies include minimizing exposure of young animals to the feces of infected adults, and reduction in environmental contamination by detection and elimination of fecal shedders (Roussel, 2011).

Johne’s disease control programs would be enhanced by a good diagnostic test for the early detection of infected animals. Various tests available for the ante-mortem diagnosis of JD are based on detection of cell mediated immunity [Jungersen et al., 2002; Huda et al., 2003; Begg et al., 2009; World Organisation for Animal Health (OIE), 2014], humoral immunity (Shin et al., 2008; Scott et al., 2010), viable MAP (Whittington et al., 2013) or detection of MAP DNA (Plain et al., 2014; Sting et al., 2014). However, most diagnostic tests for JD have poor sensitivity, particularly in the early stages of the disease, although their sensitivity increases when animals start shedding the bacteria in copious amounts (Clark et al., 2008). Poor correlation between fecal MAP load and seropositivity in ELISA has been established (Khol et al., 2012; O’Brien et al., 2013) probably owing to intermittent fecal shedding of MAP in an infected animal, although there are reports indicating that fecal shedding and seropositivity against MAP antibodies occur simultaneously (Sweeney, 2011). In general, ELISA is used for herd-level diagnosis while fecal culture and fecal polymerase chain reaction (PCR) can be used to identify individual shedders within infected herds (Diéguez et al., 2009).

Our research group recently developed the High Throughput-Johne’s (HT-J) direct fecal quantitative PCR (qPCR) test for detection of MAP DNA (Plain et al., 2014). It had an estimated specificity of 99% and sensitivities of 60% for cattle and 84% for sheep when compared to fecal culture as the reference test. HT-J qPCR has been approved for use in Australia and New Zealand for the diagnosis of bovine and ovine JD at the herd level. It may have the potential to be used for individual-animal diagnosis as it is a high throughput test, similar to the serum antibody ELISA, and like fecal culture it detects the presence of MAP. Moreover, it has higher sensitivity and specificity compared to those reported for commercially available serum antibody ELISAs. Results are available within days, unlike fecal culture which can take up to 16 weeks for confirmatory results (Gumber and Whittington, 2007; Whittington et al., 2013; Kawaji et al., 2014).

However, anecdotal evidence suggests that the HT-J qPCR test when applied for individual animal testing may not perform as well as it does for herd level testing, especially in the case of farms with high levels of MAP infection (i.e., moderate to high prevalence, heavy shedders), potentially due to PCR inhibition. It is unclear whether this is related to factors associated with the farm, such as feed or other environmental variables, or is related to individual animals, for example the stage of the disease. PCR inhibition is a well-recognized phenomenon which can adversely affect the performance of molecular diagnostic tests. The complex composition of feces might make the presence of PCR inhibitors more likely than in other types of test matrix (Monteiro et al., 1997; Thornton and Passen, 2004). Compounds such as proteins, fats, humic acid, phytic acid, Immunoglobulin G, bile, calcium chloride, EDTA, heparin and ferric chloride have been recognized as PCR inhibitors in different matrices (Rossen et al., 1992; Tsai and Olson, 1992; Al-Soud et al., 2000; Al-Soud and Rådström, 2001; Thornton and Passen, 2004). PCR inhibitors can compromise the PCR reaction and hence quantification of DNA by binding to the template DNA making it unavailable for amplification, competing with DNA for the DNA polymerase, directly inhibiting the DNA polymerase (Eckhart et al., 2000) and by competing with or chelating magnesium, an essential co-factor of the DNA polymerase (Rossen et al., 1992). However, all polymerase enzymes are not impacted equally by PCR inhibitors in the template (Al-Soud and Rådström, 2001).

There are a number of assays designed to test feces for the presence of MAP DNA (Zhang and Zhang, 2011; Park et al., 2014; Plain et al., 2014; Sevilla et al., 2014; Sting et al., 2014; Donat et al., 2015). Most of the tests designed for detection of MAP DNA in fecal samples have an internal amplification control (IAC) to differentiate between negative samples and inhibited, false negative test results. However, there are limited published data on the frequency, degree or diagnostic impact of PCR inhibition in fecal tests for Johne’s disease. Given the increasing use of PCR tests for the diagnosis of JD in general, the official status of the HT-J qPCR test in Australia and New Zealand, and anecdotal information on inhibition, this study was conducted to (i) investigate the presence of PCR inhibition in the HT-J qPCR assay in a defined sample set, (ii) partially characterize the inhibiting agent, if present, and (iii) develop strategies to identify fecal samples that may be prone to PCR inhibition so that measures could be taken for improved detection and quantification of MAP DNA.

## Materials and Methods

### Source of Fecal Samples

**FIGURE 1:**
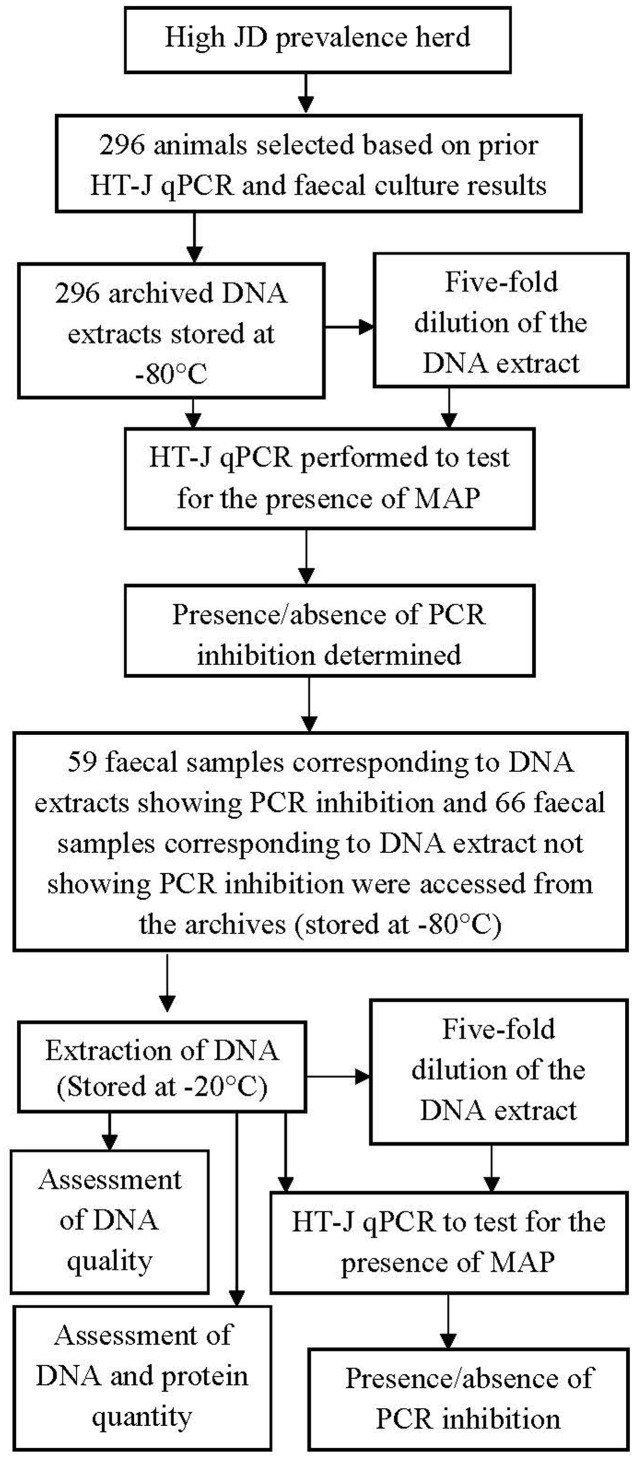
**A diagrammatic representation of the experiments**. A total of 296 archived DNA extract and the respective fecal sample were used. The result of HT-J qPCR performed on the archival DNA was used to select corresponding 125 fecal samples. Corresponding fecal culture results were also accessed post HT-J qPCR.

All animal experiments were approved by the University of Sydney Animal Ethics Committee. The fecal samples originated from two submissions from a single beef cattle herd in Tasmania, Australia known to have high prevalence of endemic JD evident clinically, microscopically and serologically. Of the total number of 2473 samples submitted, 296 fecal samples were used for this study. Based on serum antibody ELISA results, the true prevalence of infection in the herd was estimated at 45.8%; 95% CI: 30.8%, 60.8% (data not shown). The majority of the fecal samples were derived from cows >4 years old. This study consisted of two experiments; **Figure 1** shows the complete plan of the study.

### Experiment 1

This experiment was done to investigate the presence of PCR inhibition in the HT-J qPCR assay. For this experiment, 296 archived DNA extracts stored at -80°C following DNA extraction using a previously validated protocol (Plain et al., 2014) as described below in section “DNA Extraction, qPCR and PCR inhibition” of Experiment 2 and were accessed from the infectious diseases laboratory of the University of Sydney, Camden. A one-in-five dilution of the DNA extracts was performed in AVE buffer (RNase-free water with 0.04% sodium azide, Qiagen), which was used previously as the elution buffer for the extraction of DNA.

#### Fecal Culture

Fecal samples were previously cultured in BACTEC 12B liquid culture medium with PANTA PLUS, egg yolk and mycobactin J and MAP was confirmed by PCR (Whittington et al., 1998, 1999). In short, 2–5 g of feces was assorted in 10–12 mL of sterile normal saline and allowed to stand for 30 min at room temperature. Five milliliter of the surface fluid was then transferred to a tube with 25 ml of 0.9% hexadecylpyridinium chloride (HPC; Sigma Chemical Co., St. Louis, MO, USA) in half strength brain heart infusion broth (Oxoid, Basingstoke, England) and were allowed to stand at 37°C for 24 h followed by centrifugation at 900 × *g* for 30 min. The resulting pellet was suspended in 1 ml of half-strength brain heart infusion broth containing vancomycin (100 mg/ml), amphotericin B (50 mg/ml), and nalidixic acid (100 mg/ml), and was incubated at 37°C for 48 to 72 h. After incubation, 0.1 ml of the sediment was transferred to the culture vial containing Middlebrook 7H12 medium (BACTEC 12B; Becton Dickinson, Sparks, MD, USA) with 200 μl of PANTA PLUS (Becton Dickinson), 1 ml of egg yolk, 5 mg of mycobactin J (Allied Monitor Inc., Fayette, MO, USA), and 0.7 ml of sterile water. Finally, the vials were incubated at 37°C for 8 weeks. On a weekly basis, the growth index was measured using an automatic ion chamber (BACTEC 460; Johnston Laboratories, Towson, MD, USA). The growth of MAP was confirmed by *IS* 900 PCR as previously described (Whittington et al., 1998). Following extraction of the DNA using ethanol extraction method, DNA was purified by means of silica column with 6 M guanidine thiocyanate following the manufacturer’s instructions (Wizard PCR Preps DNA Purification System; Promega Corporation, Madison, WI, USA). The purified DNA was eluted in 50 ml of sterile distilled water. The PCR mixture comprised of 5 μl of purified DNA solution, 250 ng each of IS900 primers P90 (5′-GAAGGGTGTTCGGGGCCGTCGCTTAGG-3′) and P91 (5′-GGCGTTGAGGTCGATCGCCCACGTGAC-3′), 200 mM each of dATP, dTTP, dGTP, and dCTP, 66.8 mM Tris-HCl, 16.6 mM (NH4)2SO4, 2.5 mM MgCl2, 1.65 mg of bovine serum albumin per ml, 10 mM b-mercaptoethanol, and 2 U of Taq polymerase in buffer (10 mM Tris-HCl, 0.1 mM EDTA [pH 8.8]). The amplification conditions comprised of 1 cycle of denaturation at 94°C for 2 min followed by 37 cycles of denaturation at 94°C for 30 s, annealing at 62°C for 15 s, and extension at 72°C for 1 min. Visualization of the expected PCR products (400-bp) were achieved by electrophoresis at 94 V for 45 min in 2% agarose gels stained with ethidium bromide.

#### Quantitative PCR

**FIGURE 2:**
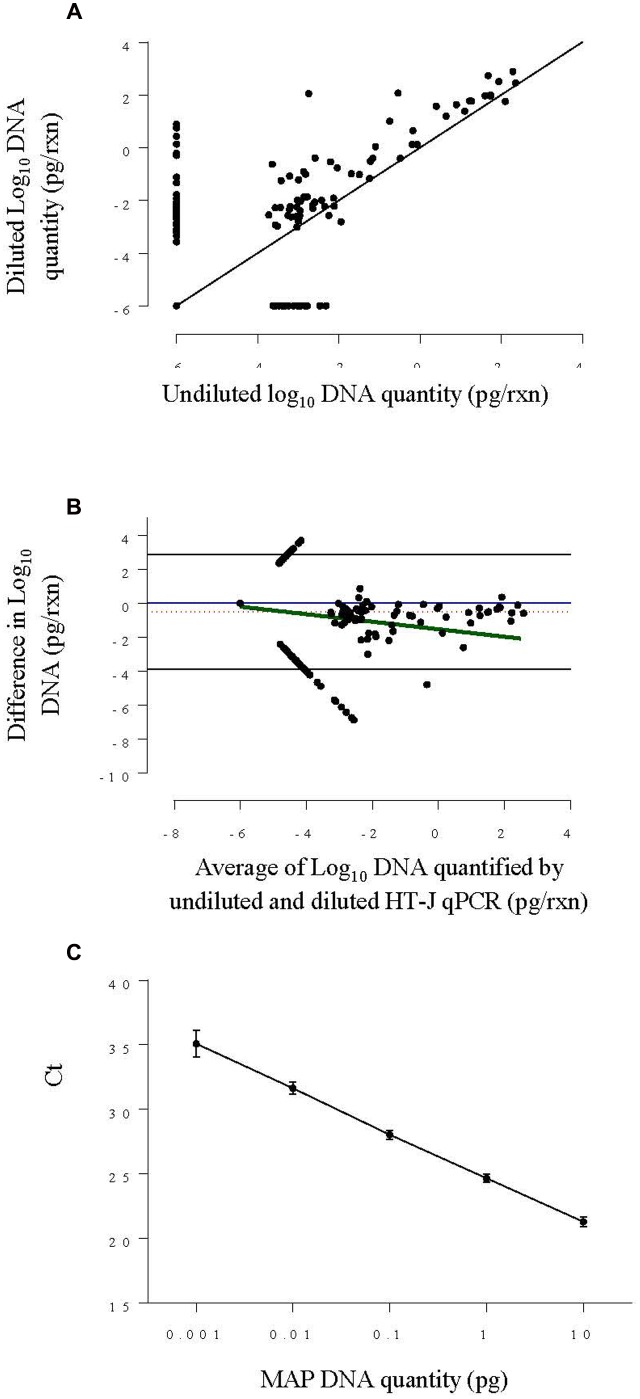
**(A)** Scatter plot of log_10_ DNA quantified by HT-J qPCR of undiluted fecal DNA extracts against diluted DNA extracts from Experiment 1. The solid black line is the ‘line of equality.’ **(B)** Bland–Altman difference plot comparing log_10_ DNA quantity of the undiluted and diluted and fecal DNA extracts from Experiment 1, by plotting the difference in MAP DNA quantified against the average DNA quantified. The solid blue line represents the optimal agreement also termed the ‘zero line’ and the region of agreement (95% confidence interval) is plotted as black lines. The dotted red line shows the average difference and the solid green line shows the regression line of the difference on the average. **(C)** Standard curve plotted by combining results from four individual standard curves constructed using 0.001–10 pg per reaction of MAP genomic DNA concentration. The point represents the mean Ct and the error bars represent ±SD of Ct.

*Mycobacterium avium* subspecies *paratuberculosis* genomic DNA was quantified in both the undiluted and the diluted DNA extracts following the HT-J qPCR method (Plain et al., 2014) which targets the *IS900* sequence of MAP. Briefly, the reaction mixtures comprised of 5 μl template DNA, 250 nM final concentration of each forward [MP10-1, (5′-ATGCGCCACGACTTGCAGCCT-3′)] and reverse [MP11-1, (5′-GGCACGGCTCTTGTTGTAGTCG-3′)] primers (Kawaji et al., 2007) and 12.5 μl of SensiMix SYBR Low-ROX qPCR mastermix (Bioline) in a final volume of 25 μl, with amplification performed using an Mx3000P real-time PCR instrument (Stratagene, Agilent). Quantification of MAP DNA was done with reference to the standard curve included in every experiment of 10-fold serially diluted MAP genomic DNA ranging from 10 to 0.001 pg/reaction. The detailed acceptance criteria for HT-J positive qPCR results was previously defined and included a cut-point of ≥0.001 pg target DNA quantity per reaction (Plain et al., 2014) equivalent to Ct value of 35.1 ± 1.04 (**Figure 2C**).

Each undiluted DNA extract was tested in a single reaction while diluted DNA extracts were tested in duplicate and a positive mean DNA test result for both replicates was considered a positive sample test result.

#### Detection of PCR Inhibition

Polymerase chain reaction inhibition was defined to be present if there was a one log increase in the DNA quantity determined from the HT-J qPCR performed on the diluted DNA extract compared to the undiluted DNA extract, after adjusting for the dilution factor by multiplication of the DNA quantity of the diluted DNA extract by five. This was due to the observation that when very low concentrations of MAP specific DNA were present in an extract, some variation in the DNA quantification is always expected, thus without an stringent cut-point, determination of PCR inhibition was not deemed to be biologically relevant.

### Experiment 2

The objective of this experiment was to partially characterize the inhibiting agent and to examine the predictability of inhibitory fecal samples. A subsample of 125 fecal samples, corresponding to 59 DNA extracts that exhibited PCR inhibition as defined above and 66 DNA extracts that did not show evidence of PCR inhibition, were accessed from the collection of archived fecal samples stored at -80°C (**Figure 1**). (Supplementary Table 1 provides additional information on the selected fecal samples).

#### DNA Extraction, qPCR and PCR Inhibition

*Mycobacterium avium* subspecies *paratuberculosis* DNA was isolated as previously described (Plain et al., 2014). Briefly, 1.2–1.5 g of feces were added to 10 ml saline to make a fecal suspension, allowed to settle for 30 min, then 3–5 ml of supernatant was centrifuged at 1231 × *g* for 30 min to obtain a pellet. Cell lysis was performed chemically using lysis buffer (Buffer RLT, BioSprint 96 one-for-all vet kit; Qiagen) and mechanically using 0.1 mm diameter zirconia/silica beads (BioSpec Products, Inc., Daintree Scientific) in a mechanical bead beater (Tissuelyser II; Qiagen) with an oscillation frequency of 30 Hz for 100 s twice after changing the alignment by 180°. DNA was purified using a magnetic bead based DNA purification kit (BioSprint 96 one-for-all vet kit; Qiagen) and platform (Biosprint-96, Qiagen). The DNA extract was diluted fivefold in AVE buffer (Qiagen). Both the undiluted and the fivefold diluted DNA extract were stored at -20°C till further use.

Quantitative PCR was conducted and PCR inhibition was detected as described in section “Quantitative PCR and Detection of PCR Inhibition of Experiment 1.

#### Assessment of Quality and Quantity of DNA Extracts

The optical density of the undiluted DNA extracts of the 125 fecal samples included in Experiment 2 was measured by spectrophotometry (Simplinano, GE) at different wavelengths (220–330 nm). Absorbance measures (*A*_230_, *A*_260_, *A*_270_, *A*_280_, and *A*_330_) were normalized by deducting the respective readings at 320 nm.

The quality of the DNA extract was assessed by three absorbance ratios (*A*_260_/*A*_280_, *A*_260_/*A*_230_, and *A*_260_/*A*_270_). DNA extracts were classified into categories based on the cut-offs that are considered to indicate the purity of the DNA extracts. An *A*_260_/*A*_280_ ratio ≥ 1.8 indicated a pure DNA extract while <1.8 was suggestive of impure DNA extracts (Gallagher and Desjardins, 2008). Likewise, an *A*_260_/*A*_230_ ratio ≥ 2 and an *A*_260_/*A*_270_ ratio ≥ 1.2 were indicative of DNA purity (Narvaez-Zapata et al., 2005). DNA was quantified using the formulae (Barbas et al., 2007):

$$\text{DNA quantity }\left(\frac{\text{ng}}{\mu l}\right)=50\times A_{260}$$

Protein concentration was calculated (Layne, 1957) as follows:

$$\text{Protein concentration }(\frac{\text{mg}}{\text{ml}})=(1.55\times A_{280})-\left(0.76\times A_{260}\right)$$

### Statistical Analysis

Descriptive analyses included summary statistics and graphical summaries for quantitative variables and frequency tables for categorical variables. Proportions and 95% confidence intervals (CI) were calculated for categorical variables.

#### Experiment 1

Polymerase chain reaction inhibition proportion and its CI were estimated using the case definition described previously.

High Throughput-Johne’s qPCR results with and without dilution were compared. Further comparisons were made by conducting Bland Altman analysis, i.e., by plotting the difference between the neat and the diluted HT-J qPCR quantities with the average of these quantities. This analysis enabled estimation of overall difference between neat and diluted qPCR DNA quantities and whether the difference varied with increase in average qPCR DNA quantity.

The sensitivity of HT-J qPCR was calculated relative to fecal culture with and without dilution. Receiver operating curve (ROC) analysis was conducted using numeric HT-J qPCR result (DNA quantity in pg) and area under the curve (AUC) was calculated to assess the discriminatory power of HT-J qPCR to differentiate fecal culture positive and negative samples. The numeric result was then categorized into negative and positive, based on the cut-point of ≥0.001 pg, and was used to calculate kappa scores of the HT-J qPCR compared to fecal culture test results.

#### Experiment 2

Polymerase chain reaction inhibition proportion and its CI were estimated based on the samples selected from Experiment 1.

Average protein quantity, DNA quantity and absorbance ratios (*A*_260_/*A*_230_ and *A*_260_/*A*_280_) were compared between inhibitory and non-inhibitory samples using two sample *t*-tests, after log transformation and/or adjusting for unequal variances, if needed. A very small value was added to each observation before log transformation in order to avoid error arising from log transformation of zero values. A Mann–Whitney test was used to test for the difference in absorbance ratio (*A*_260_/*A*_270_) between inhibitory and non-inhibitory DNA extracts as the assumption of normality was not met even after log transformation.

Logistic regression analyses were conducted to evaluate if inhibition can be predicted based on quality and quantity of DNA extracts. Absorbance ratios (*A*_260_/*A*_280_, *A*_260/_*A*_230_, and *A*_260_/*A*_270_) were categorized based on known cut-off values representing the presence or absence of contaminants. DNA quantity and protein quantity were tested as quantitative variables but since the assumption of linearity was not met, they were categorized based on medians into high and low groups representing high and low quantity of DNA and protein in the DNA extract.

A two sided P-value less than 0.05 was considered statistically significant for all analyses reported in this manuscript, unless indicated otherwise. GenStat v 16.2.11713 (VSN International Ltd., Hemel Hempstead, UK) was used to conduct all the analyses. Figures were prepared using GraphPad Prism 6 Version 6.04 (GraphPad Software, Inc., La Jolla, CA, USA).

## Results

### Experiment 1

In Experiment 1, the presence, degree and diagnostic impact of PCR inhibition was examined in fecal samples (*n* = 296) derived from a high prevalence, endemically infected beef cattle herd. The DNA extracts derived from these fecal samples were tested, either undiluted or diluted, and the qPCR results compared.

#### HT-J PCR Test Results before and after Dilution

The results from the qPCR test were interpreted as either positive or negative, based on the criteria for a positive result using the HT-J qPCR test. Of the 296 DNA extracts tested, 57 (19.3%) undiluted fecal DNA extracts and 99 (33.5%) diluted fecal DNA extracts were test-positive (**Table 1**). A parallel interpretation, using a positive HT-J qPCR test result in either the undiluted or diluted DNA extract, resulted in further increase in the number of a positive test results to 107 (**Table 2**). Comparing the results before and after dilution, it was found that 50 previously negative samples became positive post-dilution while only eight samples went from positive to negative.

**Table 1:** High Throughput-Johne’s (HT-J) quantitative PCR (qPCR) test results of both inhibitory and non–inhibitory samples before and after dilution.

HT-J qPCR test result	Inhibitory samples (*N* = 59)		Non-inhibitory samples (*N* = 237)		Total samples (*N* = 296)	
	Undiluted	Diluted	Undiluted	Diluted	Undiluted	Diluted
Positive	12	54^a^	45	45	57	99
Negative	47	5	192	192	239	197
Total	59	59	237	237	296	296

^a^*Additional 42 samples turned positive post-dilution in the inhibitory samples*.

**Table 2:** Test results and sensitivity of HT-J qPCR under different test conditions and interpretation criteria assuming fecal culture (FC) as the gold standard.

Test conditions and interpretation criteria	HT-J qPCR test results^a^					Sensitivity (%) (95% confidence interval)
	HT-J positive and FC positive	HT-J positive and FC negative	HT-J negative and FC negative	HT-J negative and FC positive	Total	
(1) Undiluted DNA extract	44	13	203	36	296	55.0 (43.47, 66.15)
(2) Diluted DNA extract (after accounting for dilution)^b^	61	38	178	19	296	76.25 (65.42, 85.05)
(3) Result of 1 and 2 used in parallel	64	43	173	16	296	80.0 (69.56, 88.11)

^a^*At cut off ≥0.001 pg DNA/qPCR reaction for positive test result.*

^b^*DNA quantified from HT-J qPCR of five-fold diluted DNA extract was multiplied by five to account for dilution of the DNA extract and the new value was used to interpret the test results*.

#### DNA Quantity before and after Dilution

When considering the DNA quantity detected by the qPCR before and after dilution, of the 296 fecal DNA extracts, 70 samples had an increase in MAP DNA quantity detected post-dilution, while 55 showed a decrease in MAP DNA quantity. In DNA extracts that showed a decrease in MAP DNA quantity detected by the HT-J qPCR post-dilution, there was a median decrease of 0.002 pg (inter-quartile range 0.195 pg), whereas in DNA extracts showing increased MAP DNA quantities post-dilution, there was a median increase of 0.002 pg (inter-quartile range 0.046 pg).

A scatter plot of the results for all DNA extracts (**Figure 2A**) showed that the diluted DNA extracts had higher DNA quantity in general. Likewise, the Bland-Altman plot (**Figure 2B**) shows that the overall difference in log_10_ DNA quantified in neat and diluted HT-J qPCR was negative, indicating a higher DNA quantity of diluted DNA extracts. This difference increased as the average of DNA quantity decreased, indicating a higher degree of variation at DNA quantities approaching the limit of quantification of the assay (0.005 pg). It is also evident from the Bland-Altman plot that, on an average, the level of DNA quantified by HT-J qPCR of the diluted DNA extracts was 3.3 times that of the undiluted DNA extracts.

#### PCR Inhibition

In total, 59/296 fecal samples (19.94%; 95% CI: 15.38%, 24.48%) showed PCR inhibition as demonstrated by an increase in DNA quantity by one log post-dilution and adjustment for dilution according to the case definition (**Table 1**). Only 12 of these inhibitory samples were originally HT-J qPCR positive and these remained positive post PCR inhibition relief. However, following dilution and subsequent relief of inhibition, 42 (89.4%) changed from a negative to a positive test result. Of the 42 DNA extracts that went from a negative to a positive test result, 27 (64.3%) had a DNA quantity that was approaching the cut-off point for a positive test result (within the range of 0.001–0.01 pg), whereas the quantity of DNA varied from 0.02 to 7.8 pg in the remainder of these DNA extracts.

#### Comparison between HT-J and fecal Culture

**FIGURE 3:**
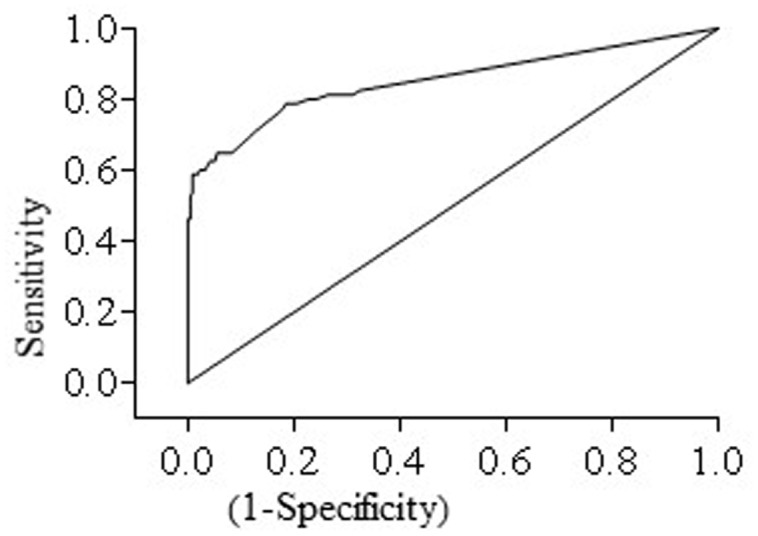
**Receiver operating curve (ROC curve) for HT-J qPCR test constructed using test results of 296 fecal samples comprising of 80 fecal culture positive and 216 fecal culture negative samples**. The area under the curve (AUC) was 0.852 (95% CI: 0.7934, 0.9108).

A total of 80 fecal samples were culture positive. Comparison between fecal culture and fecal HT-J qPCR gave a kappa score of 0.643 (95% CI: 0.540, 0.745) indicative of good agreement between the two tests. Likewise, ROC analysis gave an area under the ROC curve of 0.852 (95% CI: 0.7934, 0.9108) (**Figure 3**) indicative of the ability of HT-J qPCR to discriminate between fecal culture positive and fecal culture negative animals.

High Throughput-Johne’s qPCR on the one hand did not detect some (16) culture positive fecal samples, even after dilution, while on the other hand it detected a larger number (43) of fecal samples that tested negative by fecal culture (**Table 2**). The increased proportion of fecal culture positives that were detected in the HT-J qPCR by testing the diluted DNA extract, led to a corresponding increase in test sensitivity (**Table 2**). Considering fecal culture as the reference test, the sensitivity of the HT-J qPCR using undiluted DNA extracts from this herd was 55%, with a 95% CI of 43.47–66.15%, which is within the range originally reported for this assay of 60% (Plain et al., 2014). Dilution of the DNA extract increased the sensitivity of the HT-J qPCR to 76.25% (95% CI: 65.42, 85.05). Taking into consideration positive results from undiluted and diluted extracts (parallel test interpretation), the sensitivity of the HT-J qPCR increased to 80% (95% CI: 69.56, 88.11).

### Experiment 2

The result of Experiment 1 were used to select 125 fecal samples corresponding to 59 DNA extracts that exhibited PCR inhibition and 66 DNA extracts that did not show evidence of PCR inhibition. The fecal samples were re-extracted to examine the sample characteristics and reproducibility of inhibition.

#### PCR Inhibition

Of the 125 archival fecal samples selected from the original 296 fecal samples for secondary HT-J testing, 40 (32%) showed evidence of PCR inhibition in the second round of testing. Of the 59 archived DNA extracts initially showing inhibition according to our definition, only 17 showed inhibition in the second experiment using DNA extracts freshly prepared from the original fecal samples, while 23 of the 66 previously non-inhibitory DNA extract samples showed inhibition in the second experiment.

#### Qualitative Differences between Inhibitory and Non-inhibitory DNA Extracts

**FIGURE 4:**
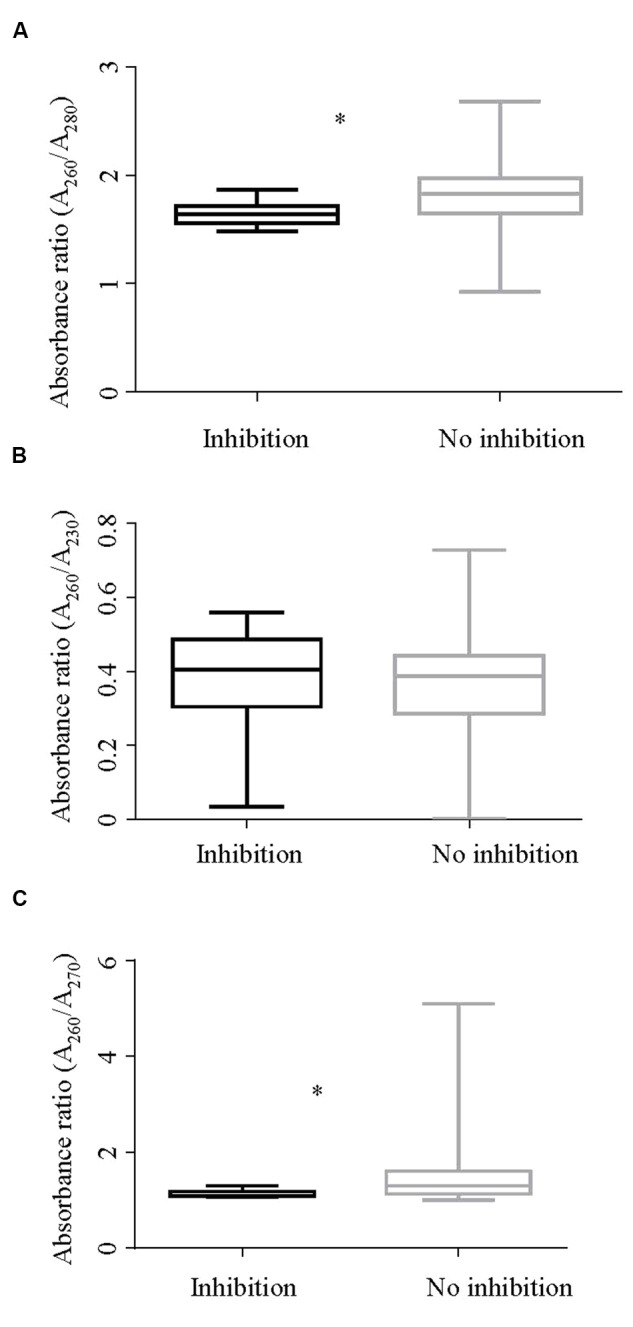
**Box and whisker plot showing absorbance ratios (A)** *A*_260_/*A*_280_, **(B)** *A*_260_/*A*_230_, and **(C)** *A*_260_/*A*_270_ by inhibition status of the DNA extracts. **(A)** Shows a significant difference (*p* < 0.001) in the *A*_260_/*A*_280_ ratios between inhibitory and non-inhibitory samples. Likewise, **(C)** shows a significant difference in rank for absorbance ratios *A*_260_/*A*_270_ of inhibitory and non-inhibitory samples in a Mann–Whitney test (*p* < 0.001). ^∗^Rrepresents significant difference.

Absorbance readings of both inhibitory and non-inhibitory DNA extracts were assessed. The summary statistics of all three absorbance ratios are presented in **Table 3**. **Figure 4** shows the descriptive summary of the three ratios. The DNA extracts showing inhibition had significantly lower mean A_260_/A_280_ ratio values when compared with those not showing inhibition as shown by *t* test. The variance of *A*_260_/*A*_280_ ratio among inhibitory and non-inhibitory samples was unequal so separate variance for each group was calculated while performing *t*-test. Likewise, a significant difference in the rank average of *A*_260_/*A*_270_ ratios of inhibitory and non-inhibitory DNA extracts was also found, with a lower rank for inhibitory DNA extracts. However, the mean *A*_260_/*A*_230_ ratio was not significantly different between inhibitory and non-inhibitory DNA extracts. Difference in variance between inhibitory and non–inhibitory samples was also noted for *A*_260_/*A*_270_ ratio. All 125 DNA extracts were free from debris indicated by an *A*_330_ value of <0.3. In contrast, all of the extracts had an *A*_260_/*A*_230_ absorbance ratio < 2 indicating the presence of contaminants in all the DNA extracts. This ratio was thus not further analyzed using logistic regression.

**Table 3:** Summary statistics of measures of quality and quantity of DNA extracts classified by inhibition status of the DNA extracts from 125 fecal samples corresponding to the selected DNA extracts from Experiment 1.

Measures of quality and quantity of DNA extract	Inhibition	*N*	Min	Q1	Median	Q3	Max	Mean ± SE	*P*
*A*_260_/*A*_280_ ratio	Yes	40	1.49	1.56	1.65	1.72	1.87	1.65 ± 0.02	<0.001^a^
	No	85	0.93	1.65	1.83	1.98	2.69	1.83 ± 0.03	
	Overall	125	0.93	1.63	1.74	1.89	2.69		
*A*_260_/*A*_270_ ratio	Yes	40	1.07	1.09	1.1	1.17	1.3	1.13 ± 0.02	<0.001^b^
	No	85	1	1.13	1.3	1.6	5.1	1.44 ± 0.06	
	Overall	125	1	1.1	1.2	1.43	5.1		
*A*_260_/*A*_230_ ratio	Yes	40	0.04	0.31	0.41	0.49	0.56	0.38 ± 0.03	0.56
	No	85	0	0.29	0.39	0.44	0.73	0.37 ± 0.02	
	Overall	125	0	0.3	0.4	0.47	0.73		
Quantity of proteins (mg/ml)	Yes	40	0.05	0.15	0.26	0.38	0.76	0.24 ± 0.03^d^	<0.001^c^
	No	85	0	0.01	0.04	0.12	0.78	0.06 ± 0.01^d^	
	Overall	125	0	0.02	0.09	0.24	0.78		
Quantity of DNA (ng/μl)	Yes	40	14	49.75	66.8	86.7	177.2	64.08 ± 5.98^d^	<0.001^a^
	No	85	4	13.4	24	44.45	144.9	24.59 ± 2.05^d^	
	Overall	125	4	18.25	31.6	65.07	177.2		

^a^*P-values from unpaired two sample t-test (variance was calculated separately for each groups of variable *A*_260_/*A*_280_ ratio).*

^b^*P-value from Mann–Whitney U test.*

^c^*P-values from unpaired t-test after log transformation of the variables (variance was calculated separately for each groups of variable protein concentration).*

^d^*dGeometric mean ± SE.*

#### Quantitative Differences between Inhibitory and Non-inhibitory DNA Extracts

**FIGURE 5:**
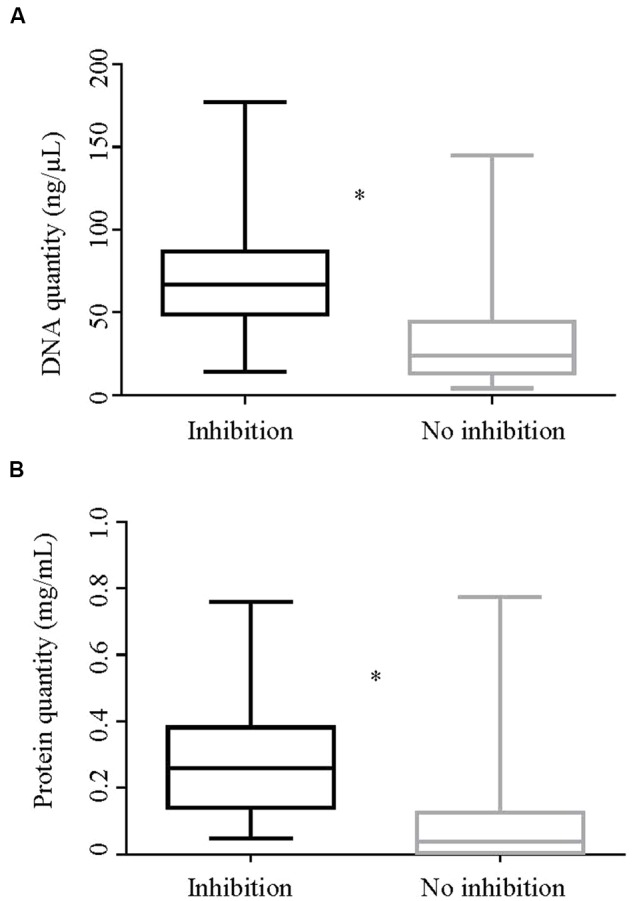
**Quantity of DNA and protein by inhibition status of the DNA extracts**. Box-and whisker plots showing **(A)**. quantity of DNA (ng/μl) and **(B)**. quantity of protein (mg/ml) of DNA extracts in Experiment 2 by their inhibition status. The geometric means of both the DNA and protein quantities between the inhibitory and non-inhibitory DNA extracts were significantly different by two sample *t*-test (*p* < 0.0001). ^∗^Represents significant difference.

DNA and protein concentrations of extracts with and without inhibition are shown in **Figure 5**. The median DNA concentration for DNA extracts showing inhibition and those not showing inhibition was 66.8 and 24 ng/mL, respectively. Likewise, the median protein concentration in DNA extracts showing inhibition was 0.258 mg/mL and for those not showing inhibition was 0.04 mg/mL. The differences in geometric mean DNA and protein concentrations between inhibitory and non-inhibitory DNA extracts were significant. The variances of log DNA concentration between inhibitory and non–inhibitory samples were equal while that of log protein was unequal.

#### Prediction of PCR Inhibition

Five variables representing quality and quantity of DNA extracts were tested in univariable logistic regression. The relationship between inhibition and DNA contamination assessed by *A*_260_/*A*_280_ and *A*_260_/*A*_270_ was significant (**Table 4**). Contamination of the DNA extract as assessed by *A*_260_/*A*_280_ ratio (<1.8) increased the odds of inhibition by 13.88 times. Likewise DNA contamination indicated by the *A*_260_/*A*_270_ ratio (<1.2) increased the odds of inhibition in the DNA extract by 6.4 times. However, no significant relationship between inhibition and fecal culture result was found (**Table 4**).

**Table 4:** Regression coefficients, odds ratio and 95% confidence interval for factors associated with PCR inhibition estimated using univariable logistic regression.

Parameters	Category	*b*	s.e (b)	Odds ratio	95% CI	*P*
Fecal culture	Positive	0.45	0.28	1.09	0.51, 2.31	0.83
	Negative	0	-	1	-	-
*A*_260_/*A*_280_ ratio	<1.8	0.07	0.60	13.88	3.97, 48.45	<0.001
	≥1.8	0	-	1	-	-
*A*_260_/*A*_270_ ratio	<1.2	0.17	0.34	6.44	2.76, 15.06	<0.001
	≥1.2	0	-	1	-	-
DNA concentration	High	0.11	0.43	10.94	4.12, 29.06	<0.001
	Low	-	-	1	-	-
Protein concentration	High	0.07	0.52	19.33	6.25, 59.76	<0.001
	Low	-	-	1	-	-

*b, regression coefficient; se, standard error; CI, confidence interval*.

DNA extracts that had high protein content had 19.33 times higher odds of showing inhibition in qPCR when compared to the DNA extracts that had a low protein concentration. Likewise, DNA extracts that had high total DNA content had 10.94 times higher odds of showing inhibition in qPCR when compared to DNA extracts that had a low total DNA concentration (**Table 4**).

## Discussion

This is the first report examining inhibition in a PCR assay for detection of MAP DNA. At least one-fifth of DNA extracts from bovine fecal samples showed some evidence of inhibition, however, variation in inhibition associated with two series of DNA extracts from the same feces was observed in this study. The possible effects of prolonged storage of fecal samples (Experiment 2) or DNA extracts (Experiment 1) at -80°C on the inhibitory substances and DNA were uncontrolled in the design. Complexity of fecal sample and non-homogeneity of subsamples may also have contributed to this observation. Furthermore, PCR inhibition is a complex phenomenon; the same inhibitory substance(s) might not always be equally inhibitory for all PCR reactions or initial sample types (Huggett et al., 2008).

Polymerase chain reaction inhibition was relieved, by a simple one-in-five dilution of the DNA extract. This approach has been applied in animal health diagnostic laboratories in Australia on an *ad hoc* basis but is not endorsed by the regulatory committee (Sub-committee on Animal Health Laboratory Standards, SCAHLS) due to lack of data on the effect on all DNA extracts. It is based on a common practice for testing other sample types such as MAP bacterial colonies in conventional PCR. Though our findings are based on samples from a beef cattle herd using a particular test platform, a similar approach may be applicable to other tests and animal cohorts. Our laboratory has successfully applied this approach to diagnostic test samples for JD from dairy cattle and various sheep breeds with similar success (unpublished data). Five-fold dilution of the DNA extract was previously found to relieve PCR inhibition in 78 to 100% of the inhibitory environmental water samples depending on the qPCR method tested (Cao et al., 2012). Relief of inhibition by dilution of the DNA extract resulted in an increase in the sensitivity of the HT-J PCR relative to fecal culture from 55 to 80% (when results for undiluted and diluted extracts were considered in parallel). The dilution factors proposed in other studies in which this phenomenon has been examined were very high (up to 1000-fold) and were dependent upon the type of DNA extraction used and the minimum inhibitory concentration of the substances present (Al-Soud et al., 2000; Chui et al., 2004). A high dilution factor eventually can undermine the detection limit of the analyte in the PCR and result in false negative results (Queipo-Ortuño et al., 2008), especially when the testing protocol is required to deal with fecal samples containing very low copy numbers of the target DNA.

This study was conducted on fecal samples sourced from a herd with a high prevalence of MAP infection and it was hypothesized that the inhibitory DNA extracts may be from high shedders with altered physiology as they progress toward clinical disease. Protein malabsorption (Allen et al., 1974b) and loss of protein into the intestines (Allen et al., 1974a,c) is the usual course in pathogenesis of JD and hypoproteinaemia is one of the signs of both bovine and ovine JD (Donat et al., 2014; McGregor et al., 2015). Assuming that there is a limit to the extent to which the DNA purification strategy excludes proteins, this could contribute higher protein concentrations in DNA extracts from fecal samples and hence PCR inhibition found in this study. Immunoglobulins could be another potential contributor to the proteins found in feces. Immunoglobulins have been noted in feces of healthy as well as diseased animals (Franz and Corthier, 1981). There is a high likelihood of the presence of both MAP-specific and non-specific immunoglobulins in feces of JD infected animals, with a mucosal immune response previously identified in JD infected cattle and sheep (Weiss et al., 2006; Begg et al., 2015). High MAP DNA quantities would be expected from the animals in an advanced stage of the disease due to high shedding rates. Although there was a several fold increase in MAP DNA quantity following relief of inhibition, the MAP DNA quantified in the inhibitory DNA extracts was not always higher than non-inhibitory DNA extracts, suggesting that the PCR inhibition phenomenon was not confined to such individuals.

The present study showed the value of spectrophotometric estimation of protein and DNA content in the DNA extract as a predictor of possible PCR inhibition in the event of a negative HT-J qPCR result. Regression analysis showed that a high content of DNA as well as protein was shown to increase the odds of inhibition. Inhibition of qPCR by non-target DNA is well documented (Tebbe and Vahjen, 1993), possibly through limiting the availability of primers and DNA polymerase to the target. The presence of host DNA has previously been reported to be inhibitory to the PCR reaction to detect MAP DNA in tissue (Radomski et al., 2013).

The presence of protein in inhibitory DNA extracts was affirmed by significantly higher odds of detecting inhibition in DNA extracts with low *A*_260_/*A*_280_ ratio, which is suggestive of contamination by molecules with peak absorbance at *A*_280,_ chiefly proteins (Gallagher and Desjardins, 2008) and other contaminants like phenol (Sik Kim et al., 2005), though it is also affected by the pH of the solution and the presence of salts (Wilfinger et al., 1997). Exogenous phenol could be discounted given that a phenol-free extraction protocol was used, however, the possible presence of polyphenolics of plant origin cannot be excluded. The *A*_260_/*A*_280_ ratio of 1.8 might not always indicate freedom of the DNA extract from protein (Glasel, 1995), but definitely suggest absence of protein in the absence of other contaminants that also give absorbance at *A*_280_ (Gallagher and Desjardins, 2008). The method of DNA purification used for this study makes it less likely for other contaminants and factors to be affecting the ratio, thereby providing a strong indication for the presence of proteins. Proteins have been reported to be capable of PCR inhibition, possibly due to coagulation; the presence of 1% pure protein (hydrolysed protein) in the PCR reaction was found to be inhibitory while 0.1% was found to be acceptable (Rossen et al., 1992). In contrast, some proteins like bovine serum albumin have been shown to relieve the inhibitory effect of humic acid when added to the DNA extract (Schriewer et al., 2011). Further investigation into the type of proteins present in the bovine fecal extracts using mass spectrometry could elucidate the mechanisms involved in the PCR inhibition exhibited in this study and identification of the compound could aid in devising a protocol to prevent PCR inhibition.

Similarly, the *A*_260_/*A*_270_ ratio could be another useful predictor of the possible inhibition state of the DNA extracts. An *A*_260_/*A*_270_ ratio of ≥1.2 is indicative of the absence of phenol, but a low *A*_260_/*A*_270_ is considered to be indicative of contamination chiefly by polyphenolics (Narvaez-Zapata et al., 2005) derived from plant-based feed.

However, all DNA extracts had low *A*_260_/*A*_230_ ratios with no significant difference between groups, suggesting presence of contaminants that have absorbance at 230 nm including contamination with contaminants like humic acid, residual guanidine, phenol, urea, polysaccharides and polycyclic aromatic hydrocarbons derived from animal feed (Narvaez-Zapata et al., 2005; Gallagher and Desjardins, 2008; Mahmoudi et al., 2011).

The fivefold dilution resulted in successful dilution of the putative contaminants in the DNA extract and led to a higher number of positive test results detected in this infected herd. This was apparent not only as an increase in the apparent sensitivity compared to fecal culture but also as an increased number of culture negative fecal samples that were HT-J positive. In terms of individual animal test outcome, only a few test positive fecal DNA extracts with a low quantity of DNA became test-negative subsequently upon dilution, indicating that the low dilution factor used might have broad application even when dealing with subclinically infected animals with a relatively low load of MAP in their feces. In contrast, an additional 20 culture positive fecal samples that were originally test negative by HT-J qPCR were detected after relief of inhibition. Fecal culture is frequently used as a reference test to assess the sensitivity and specificity of other tests for JD including ELISA (Yokomizo et al., 1983). Fecal culture has very high specificity and sensitivity compared to other ante mortem tests (Whittington et al., 2013) with higher sensitivity for liquid culture system than solid media (Whittington, 2010). The phenomenon of culture negative fecal samples that are positive by HT-J qPCR has been previously reported for this test and pertains to a number of factors including limitations of the culture method, due to the use of decontaminants that decrease the number of viable MAP in the fecal samples by several fold (Reddacliff et al., 2003), the ability of PCR to detect live as well as dead MAP organisms and stochastic sampling effects in fecal samples containing low levels of MAP (Plain et al., 2014).

The PCR inhibition control scheme adopted can vary between laboratories. The use of an IAC is one option to detect PCR inhibition and has been applied to MAP detection assays (Rodríguez-Lázaro et al., 2004). IAC could, however, compete with the template DNA if multiplexed, and may respond differently in the presence of inhibitors, dependent on differential primer and template design, demanding greater effort and resources to validate the test protocol. In addition, in the situation where the inhibition involves a mechanism other than inhibition of the DNA polymerase enzyme, IAC might not be able to facilitate detection of PCR inhibition. For example, in a study by Cao et al. (2012) the use of an IAC was found to be not as effective as fivefold dilution in detecting inhibition. Likewise, for HT-J qPCR and other test methods based on SYBR green it is not possible to multiplex with IAC.

The best way to deal with PCR inhibition may be to prevent it, which involves careful design of the DNA extraction protocol, tailor-made for a specific sample type and situation. This demands understanding of the composition of complex test matrix such as bovine feces, which can vary depending on geography, season of year, feed, physiological state of the animal, stage of the target disease, intercurrent and concurrent infections and composition of the microbial flora. The complexity of the fecal sample makes the possibility of the presence of PCR inhibitors unavoidable. Relief by dilution could be a simpler option for PCR inhibition control for the HT-J qPCR and similar tests.

## Conclusion

Polymerase chain reaction inhibition in the HT-J qPCR test was investigated for the first time in fecal samples originating from a highly JD infected herd. Investigation of more number of herds with a range of JD infection prevalence will further improve our understanding on the PCR inhibition phenomenon. If care is taken to identify and relieve PCR inhibition, the HT-J PCR method has the potential to be used as an individual animal diagnostic test. Test and relief of PCR inhibition thus should always form part of the test package if it is to be used in this setting. Absorbance measures like absorbance ratios and quantification of total DNA and protein content can be used as predictors for the identification of an inhibitory DNA extract. Therefore one recommendation may be that DNA extract be routinely tested using absorbance measures prior to qPCR so that any negative test result can be further explored if the test method does not incorporate inhibition control strategy. Alternatively, a simple fivefold dilution could be capable of both testing and relieving PCR inhibition at the same time thereby providing potential PCR inhibition control option. The choice of the methodological approach to this issue should moreover be tailored to the particular sample type, extraction method and situation. Thus, consideration of PCR inhibition should form part of molecular diagnostics to avoid consequences that can result from false-negative PCR results especially in a disease control program. This is of high merit in an insidious disease like bovine JD.

## Author Contributions

KA: Contribution to concept, planned the experiment, performed the experiment, performed analysis and interpretation of the data, drafted the manuscript and approved on the version to be submitted. ND: Contribution to concept of the work and planning of the experiment, analysis and interpretation of the data, review of the manuscript, statistical analysis and approved on the version to be submitted. RW: Contribution to concept, planning of the experiment, analysis and interpretation of the data, review of the manuscript and approved on the version to be submitted. KP: Conceived the research, contributed to the planning of the experiment, analysis and interpretation of the data, reviewed the manuscript and approved on the version to be submitted.

## Conflict of Interest Statement

The authors declare that the research was conducted in the absence of any commercial or financial relationships that could be construed as a potential conflict of interest.
