# Supplementary material for: PCR Inhibition of a Quantitative PCR for Detection of Mycobacterium avium Subspecies Paratuberculosis DNA in Feces: Diagnostic Implications and Potential Solutions
Source: Front Microbiol. 2017 Feb 2;8:115. doi: 10.3389/fmicb.2017.00115 (PMC5288348; doi:10.3389/fmicb.2017.00115)
Supplement: Supplementary file 1 [file Data_Sheet_1.DOCX]

Supplementary Material

PCR inhibition of a quantitative PCR for detection of *Mycobacterium avium* subspecies *paratuberculosis* DNA in faeces: Diagnostic implications and potential solutions

Kamal R. Acharya ^1^, Navneet K. Dhand ^1^, Richard J. Whittington^1^, Karren M. Plain ^1*^

^1^ Faculty of Veterinary Science, School of Life and Environmental Sciences, University of Sydney, 425 Werombi Road, Camden, 2570, New South Wales, Australia

*** Correspondence:**Karren M. Plain
Email: karren.plain@sydney.edu.au

# Supplementary Table

Table 1 Summary information of the selected 125 samples for Experiment 2

| Sample ID | FC | ELISA | Neat qPCR performed in 2011 (Test A) | | Neat qPCR performed in 2014 (Test B) | | Diluted qPCR performed in 2014 | | Inhibition^b^ based on | |
| --- | --- | --- | --- | --- | --- | --- | --- | --- | --- | --- |
|  |  |  | DNA quantified (pg) | HT-J test result^a^ | DNA quantified (pg) | HT-J^a^ test result^a^ | DNA quantified (pg) | HT-J test result^a^ | Test B | Test A |
| 1 | 1 | 1 | 0.020275 | 1 | 0.02026 | 1 | 0.020715 | 1 | 0 | 0 |
| 2 | 1 | 1 | 6.37 | 1 | 2.51 | 1 | 7.505 | 1 | 1 | 0 |
| 3 | 0 | 0 | 0.0010019 | 1 | 0 | 0 | 0.0012505 | 1 | 1 | 0 |
| 4 | 0 | 0 | 0 | 0 | 0 | 0 | 0.00005335 | 0 | 1 | 1 |
| 5 | 1 | 1 | 0.004018 | 1 | 0.001143 | 1 | 0.001885 | 1 | 0 | 0 |
| 6 | 0 | 0 | 0.0000881 | 0 | 0.0002381 | 0 | 0 | 0 | 0 | 0 |
| 7 | 0 | 1 | 0.00037115 | 0 | 0.000967 | 0 | 0 | 0 | 0 | 0 |
| 8 | 1 | 1 | 0 | 0 | 0 | 0 | 0.27275 | 1 | 1 | 1 |
| 9 | 1 | 0 | 0.2058 | 1 | 0.08019 | 1 | 0.21615 | 1 | 1 | 0 |
| 10 | 1 | 1 | 0.0063795 | 1 | 0.007421 | 1 | 0.0024305 | 1 | 0 | 0 |
| 11 | 1 | 1 | 0 | 0 | 0 | 0 | 0.000404 | 0 | 1 | 1 |
| 12 | 0 | 0 | 0.00037965 | 0 | 0.001178 | 1 | 0 | 0 | 0 | 0 |
| 13 | 1 | 1 | 0.007945 | 1 | 0.001369 | 1 | 0.02464 | 1 | 1 | 1 |
| 14 | 0 | 0 | 0 | 0 | 0 | 0 | 0.00028415 | 0 | 1 | 1 |
| 15 | 1 | 1 | 23.595 | 1 | 7.89 | 1 | 8.52 | 1 | 0 | 0 |
| 16 | 0 | 0 | 0.00022615 | 0 | 0 | 0 | 0.0018219 | 1 | 1 | 1 |
| 17 | 1 | 1 | 2.68 | 1 | 0.179 | 1 | 2.015 | 1 | 1 | 0 |
| 18 | 1 | 1 | 0.00070785 | 0 | 0.0003342 | 0 | 0 | 0 | 0 | 0 |
| 19 | 1 | 1 | 0.068945 | 1 | 0.006283 | 1 | 0.057605 | 1 | 1 | 0 |
| 20 | 0 | 0 | 0.0016567 | 1 | 0 | 0 | 0.0016875 | 1 | 1 | 0 |
| 21 | 1 | 1 | 0.000061855 | 0 | 0 | 0 | 0.001532 | 1 | 1 | 1 |
| 22 | 1 | 1 | 0.00189 | 1 | 0.001393 | 1 | 0.002683 | 1 | 0 | 0 |
| 23 | 1 | 1 | 0.0016375 | 1 | 0.002276 | 1 | 0.00131125 | 1 | 0 | 0 |
| 24 | 1 | 1 | 0.62825 | 1 | 0.6637 | 1 | 0.87895 | 1 | 0 | 0 |
| 25 | 1 | 1 | 0.0095215 | 1 | 0.001524 | 1 | 0.019965 | 1 | 1 | 1 |
| 26 | 1 | 1 | 0.007718 | 1 | 0.004882 | 1 | 0 | 0 | 0 | 0 |
| 27 | 0 | 0 | 0 | 0 | 0.06899 | 1 | 0.07804 | 1 | 0 | 1 |
| 28 | 0 | 0 | 0 | 0 | 0.05704 | 1 | 0.013735 | 1 | 0 | 1 |
| 29 | 0 | 0 | 0 | 0 | 0 | 0 | 0.101495 | 1 | 1 | 1 |
| 30 | 0 | 0 | 0 | 0 | 0.0006195 | 0 | 0.0166 | 1 | 1 | 1 |
| 31 | 0 | 0 | 0 | 0 | 0.005672 | 1 | 0.0005345 | 0 | 0 | 1 |
| 32 | 0 | 0 | 0 | 0 | 0.0008653 | 0 | 0.00049155 | 0 | 0 | 1 |
| 33 | 1 | 1 | 0 | 0 | 0 | 0 | 0.12255 | 1 | 1 | 1 |
| 34 | 1 | 1 | 0.00067075 | 0 | 0.001119 | 1 | 0.000806 | 0 | 0 | 0 |
| 35 | 0 | 0 | 0 | 0 | 0 | 0 | 0.0000891 | 0 | 1 | 1 |
| 36 | 0 | 0 | 0.0010629 | 1 | 0.0003626 | 0 | 0.0010806 | 1 | 1 | 0 |
| 37 | 1 | 1 | 68.235 | 1 | 87.68 | 1 | 64.66 | 1 | 0 | 0 |
| 38 | 1 | 1 | 0.000525 | 0 | 0.0009593 | 0 | 0.00203185 | 1 | 1 | 1 |
| 39 | 0 | 1 | 0 | 0 | 0 | 0 | 0.00049465 | 0 | 1 | 1 |
| 40 | 1 | 1 | 0.00003124 | 0 | 0.0006081 | 0 | 0.0009225 | 0 | 0 | 1 |
| 41 | 1 | 1 | 0.0001454 | 0 | 0 | 0 | 0.0016045 | 1 | 1 | 1 |
| 42 | 0 | 0 | 0.000037315 | 0 | 0 | 0 | 0.00015255 | 0 | 1 | 1 |
| 43 | 0 | 1 | 0.000011245 | 0 | 0 | 0 | 0.00029025 | 0 | 1 | 1 |
| 44 | 1 | 1 | 0.044335 | 1 | 0.05956 | 1 | 0.060595 | 1 | 0 | 0 |
| 45 | 1 | 1 | 0.010517 | 1 | 0 | 0 | 0.27515 | 1 | 1 | 1 |
| 46 | 1 | 1 | 51.08 | 1 | 47.7 | 1 | 107.5 | 1 | 1 | 1 |
| 47 | 1 | 1 | 0.02502 | 1 | 0.03197 | 1 | 0.01925 | 1 | 0 | 0 |
| 48 | 0 | 0 | 0.00012079 | 0 | 0 | 0 | 0.00106865 | 1 | 1 | 1 |
| 49 | 1 | 1 | 0.012915 | 1 | 0.002582 | 1 | 0.08079 | 1 | 1 | 1 |
| 50 | 1 | 1 | 0.001439 | 1 | 0.001665 | 1 | 0.0027625 | 1 | 0 | 0 |
| 51 | 1 | 1 | 0.001602 | 1 | 0 | 0 | 0.53535 | 1 | 1 | 1 |
| 52 | 1 | 1 | 0 | 0 | 0 | 0 | 0.002398 | 1 | 1 | 1 |
| 53 | 1 | 1 | 0.0052915 | 1 | 0.0002296 | 0 | 0.04698 | 1 | 1 | 1 |
| 54 | 0 | 0 | 0.0002432 | 0 | 0.0003059 | 0 | 0.0002098 | 0 | 0 | 0 |
| 55 | 0 | 0 | 0.00012315 | 0 | 0 | 0 | 0.01544 | 1 | 1 | 1 |
| 56 | 1 | 1 | 0.04555 | 1 | 0 | 0 | 1.12 | 1 | 1 | 1 |
| 57 | 1 | 1 | 0.00247935 | 1 | 0.003507 | 1 | 0 | 0 | 0 | 0 |
| 58 | 0 | 0 | 0 | 0 | 0 | 0 | 0.00027145 | 0 | 1 | 1 |
| 59 | 1 | 1 | 0.49555 | 1 | 0.6473 | 1 | 0.26945 | 1 | 0 | 0 |
| 60 | 1 | 1 | 0.0008694 | 0 | 0.0009341 | 0 | 0.001124 | 1 | 0 | 0 |
| 61 | 1 | 1 | 0.1836 | 1 | 0.3259 | 1 | 0.07874 | 1 | 0 | 0 |
| 62 | 1 | 0 | 0 | 0 | 0 | 0 | 0.0092645 | 1 | 1 | 1 |
| 63 | 0 | 0 | 0.00003489 | 0 | 0.0001878 | 0 | 0.0005605 | 0 | 1 | 1 |
| 64 | 0 | 0 | 0.000238005 | 0 | 0.0002742 | 0 | 0 | 0 | 0 | 0 |
| 65 | 0 | 0 | 0.00001119 | 0 | 0 | 0 | 0.0004898 | 0 | 1 | 1 |
| 66 | 0 | 0 | 0 | 0 | 0 | 0 | 0.00048835 | 0 | 1 | 1 |
| 67 | 1 | 1 | 0.007623 | 1 | 0.009128 | 1 | 0.0344 | 1 | 1 | 1 |
| 68 | 0 | 0 | 0.0027245 | 1 | 0.003766 | 1 | 0.0020375 | 1 | 0 | 0 |
| 69 | 1 | 0 | 0 | 0 | 0.0007625 | 0 | 0 | 0 | 0 | 0 |
| 70 | 1 | 1 | 0.0024225 | 1 | 0.002297 | 1 | 0.0009805 | 0 | 0 | 0 |
| 71 | 0 | 0 | 0.000025365 | 0 | 0 | 0 | 0.00013595 | 0 | 1 | 1 |
| 72 | 0 | 0 | 0.00047115 | 0 | 0.001048 | 1 | 0 | 0 | 0 | 0 |
| 73 | 0 | 0 | 0.000733 | 0 | 0.001048 | 1 | 0.00044895 | 0 | 0 | 0 |
| 74 | 1 | 0 | 0.000087225 | 0 | 0.0009356 | 0 | 0.00019643 | 0 | 0 | 1 |
| 75 | 0 | 0 | 0 | 0 | 0 | 0 | 0.0002233 | 0 | 1 | 1 |
| 76 | 0 | 0 | 0.00041685 | 0 | 0.0009988 | 0 | 0 | 0 | 0 | 0 |
| 77 | 0 | 0 | 0.00019625 | 0 | 0.0004689 | 0 | 0 | 0 | 0 | 0 |
| 78 | 0 | 0 | 0.000011715 | 0 | 0 | 0 | 0.0005025 | 0 | 1 | 1 |
| 79 | 1 | 1 | 0.001901 | 1 | 0.004461 | 1 | 0.0011835 | 1 | 0 | 0 |
| 80 | 1 | 1 | 32.675 | 1 | 54.42 | 1 | 18.375 | 1 | 0 | 0 |
| 81 | 0 | 0 | 0.0003352 | 0 | 0.00147 | 1 | 0 | 0 | 0 | 0 |
| 82 | 0 | 0 | 0 | 0 | 0.001664 | 1 | 0 | 0 | 0 | 0 |
| 83 | 1 | 0 | 0.00012943 | 0 | 0 | 0 | 0.0033185 | 1 | 1 | 1 |
| 84 | 1 | 0 | 0.0003299 | 0 | 0.0006793 | 0 | 0.00047335 | 0 | 0 | 0 |
| 85 | 0 | 0 | 0.0002583 | 0 | 0.0002722 | 0 | 0.001043 | 1 | 1 | 1 |
| 86 | 0 | 0 | 0.0001254 | 0 | 0.0004467 | 0 | 0 | 0 | 0 | 0 |
| 87 | 0 | 0 | 0.0003221 | 0 | 0.0001938 | 0 | 0.000564 | 0 | 1 | 0 |
| 88 | 1 | 1 | 82.91 | 1 | 194.9 | 1 | 153.3 | 1 | 0 | 0 |
| 89 | 1 | 1 | 1.98 | 1 | 4.36 | 1 | 3.11 | 1 | 0 | 0 |
| 90 | 1 | 0 | 0.0001439 | 0 | 0 | 0 | 0.003321 | 1 | 1 | 1 |
| 91 | 0 | 0 | 0 | 0 | 0.0005618 | 0 | 0.000533 | 0 | 0 | 1 |
| 92 | 0 | 0 | 0 | 0 | 0 | 0 | 0.0007202 | 0 | 1 | 1 |
| 93 | 0 | 1 | 2.4255E-06 | 0 | 0 | 0 | 0.0005885 | 0 | 1 | 1 |
| 94 | 1 | 1 | 0.9877 | 1 | 0.2868 | 1 | 23.305 | 1 | 1 | 1 |
| 95 | 1 | 1 | 9.115 | 1 | 40.13 | 1 | 18.565 | 1 | 0 | 1 |
| 96 | 1 | 1 | 0.0016795 | 1 | 0.007657 | 1 | 0.0012185 | 1 | 0 | 0 |
| 97 | 0 | 0 | 0.00028895 | 0 | 0.001006 | 1 | 0.0003359 | 0 | 0 | 0 |
| 98 | 1 | 1 | 0.1758 | 1 | 0.8511 | 1 | 0.26645 | 1 | 0 | 0 |
| 99 | 0 | 0 | 0 | 0 | 0 | 0 | 0.001187 | 1 | 1 | 1 |
| 100 | 0 | 0 | 0.00004233 | 0 | 0.0002766 | 0 | 0.0002356 | 0 | 0 | 1 |
| 101 | 1 | 0 | 0.0002902 | 0 | 0 | 0 | 0.00054 | 0 | 1 | 0 |
| 102 | 0 | 0 | 0.0001015 | 0 | 0.00041 | 0 | 0 | 0 | 0 | 0 |
| 103 | 1 | 1 | 4.96 | 1 | 16.68 | 1 | 11.62 | 1 | 0 | 1 |
| 104 | 1 | 0 | 0.0002013 | 0 | 0.001025 | 1 | 0.0121455 | 1 | 1 | 1 |
| 105 | 1 | 1 | 2.205 | 1 | 12.67 | 1 | 4.85 | 1 | 0 | 1 |
| 106 | 0 | 0 | 0 | 0 | 0 | 0 | 0.00011835 | 0 | 1 | 1 |
| 107 | 1 | 1 | 4.75 | 1 | 18.51 | 1 | 11.73 | 1 | 0 | 1 |
| 108 | 0 | 0 | 0 | 0 | 0 | 0 | 0.00023135 | 0 | 1 | 1 |
| 109 | 0 | 0 | 0.00014485 | 0 | 0.0003782 | 0 | 0.011208 | 1 | 1 | 1 |
| 110 | 1 | 1 | 0 | 0 | 0 | 0 | 1.56 | 1 | 1 | 1 |
| 111 | 1 | 0 | 0.001993 | 1 | 0.01144 | 1 | 0.00031495 | 0 | 0 | 0 |
| 112 | 0 | 0 | 0.00008912 | 0 | 0.000573 | 0 | 0 | 0 | 0 | 0 |
| 113 | 0 | 0 | 0.00057175 | 0 | 0.001094 | 1 | 0.00053385 | 0 | 0 | 0 |
| 114 | 1 | 1 | 5.125 | 1 | 0.00179 | 1 | 22.685 | 1 | 1 | 1 |
| 115 | 0 | 0 | 0.0000991 | 0 | 0.0003405 | 0 | 0 | 0 | 0 | 0 |
| 116 | 1 | 1 | 0.0016155 | 1 | 0 | 0 | 0.0155 | 1 | 1 | 1 |
| 117 | 1 | 1 | 19.08 | 1 | 55.62 | 1 | 19.505 | 1 | 0 | 0 |
| 118 | 1 | 1 | 26.14 | 1 | 126.4 | 1 | 11.465 | 1 | 0 | 0 |
| 119 | 0 | 0 | 0.00009751 | 0 | 0.0002251 | 0 | 0 | 0 | 0 | 0 |
| 120 | 0 | 1 | 0.0008278 | 0 | 0 | 0 | 0.0008782 | 0 | 1 | 0 |
| 121 | 0 | 0 | 0.00040645 | 0 | 0.003425 | 1 | 0 | 0 | 0 | 0 |
| 122 | 1 | 0 | 0.00015065 | 0 | 0.001491 | 1 | 0 | 0 | 0 | 0 |
| 123 | 0 | 1 | 0.00055435 | 0 | 0.002536 | 1 | 0.001719 | 1 | 0 | 1 |
| 124 | 0 | 0 | 0.00071575 | 0 | 0.0006327 | 0 | 0.0011645 | 1 | 0 | 0 |
| 125 | 1 | 1 | 219.9 | 1 | 226.5 | 1 | 57.485 | 1 | 0 | 0 |

1. HT-J test positive (1) or negative (0) based on the definition provided in the Methods.
2. Inhibition evident (1) or not evident (0) based on the case definition provided in Methods.
